# Effect of Piperine Codelivery on the Oral Bioavailability of Cannabidiol: Insights from *In Vitro* Digestion and *In Vivo* Pharmacokinetics

**DOI:** 10.1021/acs.jafc.6c02678

**Published:** 2026-06-18

**Authors:** Renata Vardanega, Fernanda L. Lüdtke, Luis Loureiro, Andrea Fernández-Carrera, Joana Santos, Armando Venâncio, Ana C. Pinheiro, África González-Fernández, António A. Vicente

**Affiliations:** † Centre of Biological Engineering, 56059 University of Minho, Braga 4710-057, Portugal; ‡ LABBELS-Associate Laboratory, Guimarães 4800-058, Portugal; § CINBIO, 16784 University of Vigo, Vigo 36310, Spain; ∥ CINBIO, Immunology Group, University of Vigo, Vigo 36310, Spain; ⊥ Immunology, Instituto de Investigación Sanitaria Galicia Sur (IIS-GS), SERGAS-UVIGO, Vigo 36312, Spain

**Keywords:** pharmacokinetics, bioavailability, bioenhancer, lipid-based systems, cannabidiol, piperine

## Abstract

This study investigated whether coencapsulation of cannabidiol (CBD) and piperine (PIP) in food-grade nanostructured lipid carriers (NLCs) enhances the CBD oral bioavailability. NLCs containing long-chain fatty acids were formulated with 1% CBD and 1% PIP using either a CBD isolate (CBD_iso_) or a CBD extract (CBD_ext_). Both systems showed high encapsulation efficiency and good stability over 28 days, with similar physicochemical properties. However, the CBD form strongly influenced digestion behavior: NLC-PIP-CBD_iso_ exhibited high bioaccessibility for CBD (87 ± 5%), while NLC-PIP-CBD_ext_ showed markedly lower values (13 ± 4%). Based on these results, NLC-PIP-CBD_iso_ was evaluated in a mouse pharmacokinetic study against NLC-CBD_iso_ and CBD in hemp seed oil. While NLC-CBD_iso_ did not improve absorption, NLC-PIP-CBD_iso_ doubled the CBD systemic exposure, confirming that PIP codelivery significantly enhanced oral CBD absorption.

## Graphical abstract

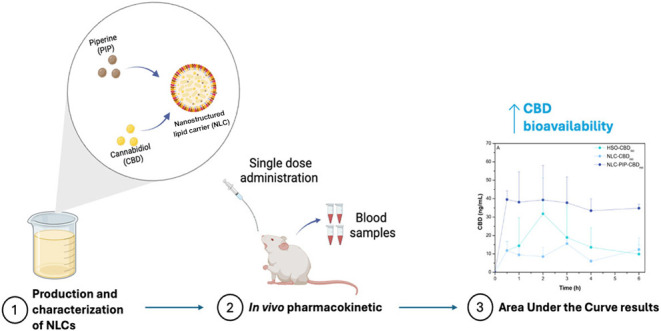

## 1 Introduction

Cannabis (*Cannabis sativa*) is one of the most ancient crops cultivated for diverse purposes, including its use as a source of food, textile fibers, and animal feed.^1,2^ It is recognized as a crop resistant to pests and drought, and capable of protecting soil layers from erosion, which makes it a promising species for widespread and sustainable cultivation.^3^ Despite these valuable attributes, cannabis cultivation has been prohibited in most countries for decades, primarily due to its psychoactive effects attributed to the presence of Δ^9^-tetrahydrocannabinol (Δ^9^-THC), the major active cannabinoid of the plant.^4,5^ Alongside Δ^9^-THC, cannabidiol (CBD) is one of the most prominent cannabinoids, widely studied for its pharmacological potential,^6,7^ including the treatment of pain in adults, chemotherapy-induced nausea and vomiting, and spasticity associated with multiple sclerosis.^8^

The renewed scientific interest in cannabinoids, driven by the evidence of their therapeutic potential, particularly for CBD and Δ^9^-THC, combined with continuous changes in the regulatory restrictions of their medical and recreational use, has sparked a rapid expansion of the cannabis-based products market.^9^ Among these, food products represent one of the fastest-growing segments, with market growth expected to continue in the coming years.^3,10^ Nevertheless, incorporating cannabinoids into food products remains a technological challenge, requiring strategies to ensure safe, effective, and consistent formulations.^1^

A major limitation in formulating cannabinoid-enriched foods is the inherently low water solubility of CBD, which restricts its incorporation into aqueous-based food products and contributes to its low oral bioavailability.^11,12^ This issue is compounded by extensive first-pass hepatic metabolism, which significantly reduces the fraction of cannabinoids reaching the systemic circulation.^13,14^ These pharmacokinetic limitations drive substantial intra- and intersubject variability in humans, making it difficult to predict the physiological effects in consumers.^11^ Furthermore, emerging evidence suggests that cannabinoid bioavailability is modulated by gender, with higher levels observed in females than in males.^15,16^ However, the mechanisms underlying this difference remain unclear, as the effect does not appear to be directly associated with the body weight differences between males and females.^15,16^ Interestingly, Knaub^16^ found that incorporating CBD into a self-nanoemulsifying drug delivery system (SEDDS) not only increased oral CBD bioavailability but also minimized these gender-based differences compared with MCT oil. Thus, improving CBD bioavailability through advanced delivery systems may represent a viable strategy to reduce pharmacokinetic variability and provide more consistent consumer effects.

A wide variety of lipid-based delivery systems have been explored to encapsulate cannabinoids, offering promising strategies to address their poor water solubility and limited bioavailability.^9^ Among these, nanostructured lipid carriers (NLCs) have emerged as a particularly effective approach for facilitating CBD incorporation into aqueous-based food matrices and enhancing its oral bioavailability.^7,12,17,18^ NLCs, comprising a mixture of solid and liquid lipids dispersed in an aqueous phase stabilized by an emulsifier, are considered the second generation of lipid nanoparticles, capable of effectively entrapping active ingredients.^12,19^ Their physicochemical characteristics confer several advantages, including enhanced stability, high loading capacity, and protection of sensitive compounds.^20^ Moreover, NLCs can improve the bioavailability of lipophilic compounds such as CBD, since coingestion with lipids, particularly those containing long-chain fatty acids, promotes lymphatic absorption and prevents extensive first-pass metabolism.^21^

Another promising strategy to enhance cannabinoid bioavailability involves the coadministration of natural absorption enhancers, also called bioenhancers.^22^ Some alkaloids, flavonoids, and several phenolic compounds can act as bioenhancers by inhibiting the action of major drug-metabolizing enzymes of the cytochrome P450 (CYP450) family, P-glycoprotein efflux pumps, and UDP-glucuronyl-transferases.^23–25^

Piperine (PIP), a natural alkaloid predominantly found in black pepper (*Piper nigrum* L.), has been extensively reported as a potential bioavailability enhancer for lipophilic compounds.^26,27^ Cherniakov et al.^22^ successfully developed a self-nanoemulsifying drug delivery system (SNEDDS) incorporating PIP and evaluated its effect on the CBD and THC bioavailability. Their findings revealed that SNEDDS containing both PIP and CBD achieved a 6-fold increase in the area under the curve (AUC) compared with a CBD solution, while the SNEDDS containing PIP and THC achieved a 9.3-fold increase compared with a THC solution, demonstrating that PIP can effectively enhance the systemic availability of these cannabinoids.

We hypothesized that the simultaneous delivery of CBD and PIP coencapsulated within an NLC formulated with lipid matrices rich in long-chain fatty acids would significantly enhance CBD bioavailability. This improvement is expected to result from the combined effects of increased lymphatic absorption promoted by long-chain fatty acids and the inhibition of first-pass metabolism mediated by PIP acting as a bioenhancer. Thus, NLCs were loaded with CBD in both forms, isolate (CBD_iso_) and extract (CBD_ext_), and PIP (NLC-PIP-CBD_iso_ and NLC-PIP-CBD_ext_) to evaluate their physicochemical characteristics as well as their performance in *in vitro* digestion and *in vivo* pharmacokinetic studies.

## 2 Materials and Methods

### 2.1 Materials

The liquid and solid lipids used to produce the NLCs were hemp seed oil (HSO) (Natursoy, Barcelona, Spain) and fully hydrogenated soybean oil (FHSO) (Cargill Foods, São Paulo, Brazil). The emulsifier used was soybean lecithin (SOLEC, Solae, Esteio, Brazil). Isolate CBD (CBD_iso_, 99.2%) and broad-spectrum CBD distillate (CBD_ext_, 87.0%) were provided by Essentia Pura (Ljubljana, Slovenia), and PIP was purchased from Sigma-Aldrich (Saint-Louis, USA). Analytical standards for CBD and PIP, as well as the internal standard CBD-d3, were provided by Sigma-Aldrich (Saint-Louis, USA). Acetonitrile (HPLC grade) and *n*-hexane (HPLC grade) were purchased from Fisher Chemical (Hampton, USA). Formic acid (99–100%) and hydrochloric acid were provided by Chem-Lab (Zedelgem, Belgium).

The reagents used for *in vitro* digestion, including pepsin from porcine gastric mucosa, bile extract porcine, pancreatin from porcine pancreas (8 × USP), as well as the salts used to prepare oral, gastric, and intestinal electrolyte solutions, Perfabloc SC, sodium hydroxide (NaOH), and Nile Red, were purchased from Sigma-Aldrich (Saint-Louis, USA).

The substances used for the pharmacokinetic study were isoflurane (Isoflo, Ecuphar, Barcelona, Spain), ketamine (Anesketin 100 mg/mL, Dechra Veterinary Products, Barcelona, Spain), medetomidine (Domtor 1 mg/mL, Ecuphar, Barcelona, Spain), and sodium heparin (20 UI/mL, Rovi, Madrid, Spain).

### 2.2 NLC Production

NLCs were prepared using a previously optimized formulation consisting of HSO:FHSO (60:40, w/w) as the lipid phase (10%, w/w) and soybean lecithin as the emulsifier (3%, w/w).^17^ To produce the NLCs, the lipid phase was added to the emulsifier, and the active compounds (CBD_iso_, CBD_ext_, and PIP) at 1% (w/w) concentration were heated until complete solubilization of all components, followed by the addition of the aqueous phase preheated at 85 °C. This mixture was homogenized for 5 min at 7,000 rpm (Ultra-Turrax T18, Ika-Werke, Staufen, Germany) and then subjected to high-intensity ultrasonication (Vibra-cell VCX 500, 20 kHz, Sonics & Materials, Newtown, USA) at 40% amplitude for 4 min with pulses (4 s on, 2 s off). The concentration of the CBD and PIP loaded into the NLCs was defined as 1% for both compounds based on the loading capacity of the NLC (data not published) and previous studies that evaluated the ratios of different bioactive compounds and PIP between 1.5:1 and 1:2 (w/w).^22,28^

### 2.3 Nanostructure Characterization

#### 2.3.1 Particle Size, Polydispersity Index, and Zeta Potential

The particle size (PS), polydispersity index (PDI), and zeta potential (ZP) of the NLCs were monitored over 30 days of storage at 4 °C, using dynamic light scattering (Zetasizer Nano SZ, Malvern, Worcestershire, U.K.). For the analysis, the samples were diluted 1:100 (v/v) using ultrapure water for the fresh NLCs. PS and ZP were also measured in the samples resulting from *in vitro* digestion. These samples were diluted 1:10 (v/v) with ultrapure water for the oral, intestinal, and micellar phase samples, and ultrapure water adjusted to pH 3.0 for the gastric phase samples. The oil refractive index and particle absorbance values used in the Malvern software were 1.47 and 0.001, respectively. The mean PS was expressed as the z-average (nm).

#### 2.3.2 Entrapment Efficiency

The entrapment efficiency (EE) was measured as previously described.^17^ The amount of nonencapsulated CBD and PIP was quantified after separating the free fraction by ultrafiltration/centrifugation using Amicon ultrafiltration devices (100 kDa cutoff, Millipore, Burlington, USA). For analysis, the NLCs were diluted 1:50 in ultrapure water and transferred to the upper chamber of the Amicon device, followed by centrifugation at 4000 rpm for 30 min (Mikro 120, Hettich Centrifuge, Tuttlingen, Germany). The filtrated phase, corresponding to the unentrapped compounds, was analyzed by a Ultra-High-Performance Liquid Chromatography-Diode Array Detector (UHPLC-DAD) (Nexera X2, Shimadzu, Kyoto, Japan) following the method described by De Pra et al.^29^ CBD and PIP were identified at 220 and 343 nm, respectively, and quantification was performed using external calibration curves in the range of 1 to 50 μg/mL (*r* ^2^ = 0.998 for both compounds). EE was calculated using eq 1

1 $$\mathrm{EE}\;\left(\%\right)=\left(\frac{\mathrm{total}\;\mathrm{compound}-\mathrm{free}\;\mathrm{compound}}{\mathrm{total}\;\mathrm{compound}}\right)\times 100$$

### 2.4 *In Vitro* Digestion

The INFOGEST harmonized protocol^30^ was applied for the *in vitro* digestion evaluation of the NLCs using 5 mL of the sample in triplicate. The compositions of the simulated salivary (SSF), gastric (SGF), and intestinal (SIF) fluids are presented in Table S1. *In* *vitro* digestion began with the oral phase, in which SSF, 0.3 M CaCl_2_(H_2_O)_6_, and ultrapure water were mixed with the sample and incubated at 37 °C for 2 min under agitation (RotoTherm Plus, H2024-E-UK, Benchmark Scientific, Sayreville, USA). For the gastric step, SGF, 0.3 M CaCl_2_(H_2_O)_6_, and pepsin solution (ensuring a final activity of 2000 U/mL) were added. The pH of the mixture was adjusted to 3.0 with HCl 1 M, completed to the required volume with ultrapure water, and then incubated at 37 °C for 2 h under continuous agitation. Finally, the intestinal phase was followed by adding SIF, 0.3 M CaCl_2_(H_2_O)_6_, bile salts (10 mM in the final mixture), and pancreatin solution to achieve a final activity of 100 U/mL. The pH was then adjusted to 7.0 using either NaOH 1 M or HCl 1 M. After adjusting the final volume with ultrapure water, the sample was incubated again at 37 °C for 2 h under agitation. To terminate digestion, Perfabloc (1 mM) was added at a ratio of 10 μL per mL of sample. The final digestion mixture, also called the digesta phase, was centrifuged at 18,500*g* at 4 °C for 30 min (Multifuge XR3, Thermo Fisher Scientific, Waltham, USA), and the supernatant was collected as the micellar fraction. The oral, gastric, digesta, and micellar samples were subsequently characterized for PS, ZP (as described in Section 2.3.1), and microstructure (Section 2.4.1).

#### 2.4.1 Fluorescence Microscopy

The nanostructures before *in vitro* digestion and the samples collected after each digestion stage were examined by fluorescence microscopy using a BX51 microscope (Olympus, Tokyo, Japan), following the method described by Gonçalves et al.^31^ Prior to visualization, the samples were stained with Nile Red, applying the dye at a 1:10 (v/v) ratio relative to the sample.

#### 2.4.2 CBD Bioaccessibility, Stability, and Estimated Bioavailability

Digesta and micellar samples were assessed according to the procedure described by Vardanega et al.^32^ to evaluate CBD bioaccessibility. For the extraction step preceding CBD quantification, 200 μL of either the digesta or micellar samples were combined with 2.5 mL of *n*-hexane, vortexed for 30 s, and centrifuged for 10 min at 6000 rpm (Mikro 120, Hettich Centrifuge, Tuttlingen, Germany) at 25 °C. The upper organic phase was carefully collected in a 5 mL volumetric flask. The remaining aqueous phase was re-extracted twice using 1.0 mL of *n*-hexane per cycle, and all organic fractions were combined in a 5 mL volumetric flask before completing the volume with *n-*hexane. An aliquot of this extract was filtered and analyzed by UHPLC for CBD quantification, as described in Section 2.3.2. The CBD bioaccessibility, stability, and estimated bioavailability were subsequently calculated using eqs 2–4.

2 $$\mathrm{bioaccessibility}\;\left(\%\right)=\left(\frac{C_{\mathrm{CBD}\;\mathrm{micelle}}}{C_{\mathrm{CBD}\;\mathrm{digesta}}}\right)\times 100$$

3 $$\mathrm{stability}\;\left(\%\right)=\left(\frac{C_{\mathrm{CBD}\;\mathrm{digesta}}}{C_{\mathrm{CBD}\;\mathrm{initial}}}\right)\times 100$$

4 estimatedbioavailability(%)=bioaccessibility×stability

where *C*_CBD micelle_ and *C*_CBD digesta_ correspond to the CBD concentrations quantified in the micellar and digesta fractions, respectively. *C*_CBD initial_ represents the amount of CBD present in the nanostructures prior to the start of the digestion process.

### 2.5 Free Fatty Acid Release

The release of free fatty acids (FFA) was assessed following the procedure of Pinheiro et al.^33^ with minor modifications. The oral and gastric steps of the simulated digestion phases were carried out exactly as described in Section 2.4. The sample collected at the end of the gastric phase was combined with all the solutions of the intestinal phase (except the pancreatin solution), and the pH was adjusted to 6.9 using either 1 M HCl or 1 M NaOH. After this, the pancreatin solution was added, and the pH was maintained at 7.0 by continuously adding 0.05 M NaOH solution using a pH-stat titration temperature-controlled reactor at 37.0 ± 0.5 °C with constant stirring. Following this period, the titration setpoint was shifted to pH 9.0 and held for an additional 30 min to guarantee full ionization and titration of FA. Control assays without pancreatin were performed to determine the NaOH volume required to reach pH 7.0 in the absence of enzymatic activity. The percentage of FFA released was determined using eq 5.^34^

5 $$\mathrm{FFA}\;\left(\%\right)=\left(\frac{\left(V_{\mathrm{NaOH}\;\mathrm{sample}}-V_{\mathrm{NaOH}\;\mathrm{blan}k}\right)\times m_{\mathrm{NaOH}}\times {\mathrm{ME}}_{\mathrm{lipid}}}{w_{\mathrm{lipid}}\times 2}\right)\times 100$$

where *V*_NaOH sample_ and *V*_NaOH blank_ correspond to the volumes of NaOH consumed during the titration of the sample and blank, respectively. *m*_NaOH_ represents the molar concentration of the NaOH solution (0.05 M). ME_lipid_ refers to the lipid equivalent molecular weight (87.64 g/mol), calculated based on the FFA profiles of HSO^35^ and FHSO.^36^ Finally, *w*_lipid_ is the initial mass of the lipid (g) present in the system.

### 2.6 *In Vivo* Pharmacokinetics

#### 2.6.1 Animals and Experimental Protocol

The *in vivo* pharmacokinetic study was conducted at the Bioexperimentation Service of the University of Vigo (Spain), and the experimental protocol was approved by the Ethical Committee of Animal Experimentation of the University of Vigo (number ES360570215601/22/FUN.01/FARM.03/C/AGF02). Male and female Swiss mice (*Mus musculus*) aged between 8 and 12 weeks were maintained in cages (5 animals per cage) at ±25 °C and relative humidity of 55 ± 10%, with *ad libitum* access to food and water for 1 week for acclimatization.

The samples evaluated in this study were the CBD control (CBD_iso_ in HSO at a concentration of 1%, named as HSO-CBD_iso_), NLC-CBD_iso_, and NLC-PIP-CBD_iso_. The animals (*n* = 5) were divided into 10 groups (5 female and 5 male) containing 10 animals per group. A 100 μL dose of each sample was administered to the animals by oral gavage following the administration of inhalatory analgesia (isoflurane). This volume was the maximum established in the protocol approved by the Ethical Committee, corresponding to a dose of 50 mg/kg of both compounds, which is within the range reported in the literature.^22,28,37^ Blood samples were collected at 0, 0.5, 1, 2, 4, and 6 h, following the administration (Table S3). Each animal was subjected to a maximum of three collection times, and the first two collection points were via the mandibular vein, while the endpoint was collected via terminal cardiac punch, as detailed in Table S2. For the final cardiac puncture, an anesthetic mixture containing ketamine (75 mg/kg) and medetomidine (1 mg/kg) was administered via an intraperitoneal injection.

#### 2.6.2 Blood Sample Preparation and Compound Quantification

Blood samples collected in heparinized tubes were centrifuged at 4000 rpm for 15 min at 4 °C using an Eppendorf 5437R centrifuge (Hamburg, Germany) to obtain plasma. Subsequently, 25 μL of the internal standard (CBD-*d*_3_, 100 ng/mL) and 100 μL of methanol were added to 50 μL of plasma. The mixture was vortexed for 30 s and centrifuged at 14,000 rpm for 5 min. The resulting supernatant was then brought to a final volume of 500 μL with methanol prior to analysis.

For the analysis of the samples, a Vanquish Flex UHPLC system coupled to an Orbitrap Exploris 120 high-resolution accurate mass spectrometer (Thermo Fisher Scientific, Bremen, Germany) was used. Chromatographic separation was achieved using a Kinetex C18 column (150 mm × 4.6 mm i.d., 2.6 μm, Phenomenex, Torrance, USA). The mobile phases consisted of water (A) and acetonitrile (B), both containing 0.1% formic acid. Elution was performed under gradient conditions at a flow rate of 0.5 mL/min as follows: 0–1 min, 25% B; 1–2 min, 75% B; 2–5 min, 75% B; 5–6, 25% B; 6–10 min, 25% B. The column was maintained at 40 °C, the autosampler at 15 °C, and the injection volume was 10 μL. Mass spectrometric detection was performed using a heated electrospray ionization (HESI) probe (OptaMax NG, Thermo Fisher Scientific, Bremen, Germany). External mass calibration of the Q-Orbitrap was performed weekly to maintain mass accuracy below 3 ppm. The Orbitrap Exploris 120 mass spectrometer was equipped with a HESI source. The HESI parameters were as follows: source temperature, 350 °C, capillary temperature, 325 °C, electrospray voltage, 3.5 kV in positive mode and 2.5 kV in negative mode. Sheath and auxiliary gas were 50 and 10, respectively. Instrument operation and data processing were performed using Xcalibur 4.5 software (Thermo Fisher Scientific, Bremen, Germany).

### 2.7 Pharmacokinetic and Statistical Analyses

All analyses were performed in triplicate, and the data are presented as mean ± standard deviation (SD). The pharmacokinetic evaluation included the determination of maximum plasma concentration (*C*_max_), time to reach the maximum concentration (*T*_max_), and the area under the curve (AUC), calculated using the trapezoidal log/linear method. Differences among groups were assessed using one-way analysis of variance (ANOVA), followed by Tukey’s test. Statistical difference was defined at 95% (*p*-value ≤ 0.05), and all statistical procedures were performed using Minitab 20.0 software (State College, USA).

## 3 Results and Discussion

### 3.1 NLC Characterization and Stability

Figure 1a–c present the PS, PDI, and ZP of the NLCs loaded with PIP+CBD_ext_ (NLC-PIP-CBD_ext_) and PIP+CBD_iso_ (NLC-PIP-CBD_iso_) during the storage time (i.e., 28 days). These systems loaded with both active compounds, CBD and PIP, presented a PS of around 220 nm during the whole period (Figure 1a), similar to that previously reported for NLCs loaded with only CBD_ext_ or CBD_iso_, which presented a PS in the range of 218.4 ± 0.1–231.5 ± 3.5 nm for NLC-CBD_iso_ and 207.7 ± 1.2–214.8 ± 1.2 nm for NLC-CBD_ext_.^17^ These results suggest that the addition of PIP to the systems did not significantly affect the formation of the NLC structure. The PDI of the systems was around 0.210 and 0.230 for NLC-PIP-CBD_ext_ and NLC-PIP-CBD_iso_, respectively (Figure 1b), which were even below the values observed for NLCs loaded with only CBD in the range of 0.250.^17^ These results indicate a narrow size distribution and excellent stability over storage time. The ZP of both systems presented a more pronounced variation during the storage time, particularly for NLC-PIP-CBD_iso_, which presented a value of −43 ± 2 mV for the fresh sample, followed by a significant decrease after 7 days of storage (−62.3 ± 0.8 mV), and then reaching −46 ± 1 mV after 28 days of storage. NLC-PIP-CBD_ext_ presented values of around −58 ± 2 mV for the freshly prepared sample, which decreased to −46 ± 1 mV after the storage period (Figure 1c).

**Figure 1:**
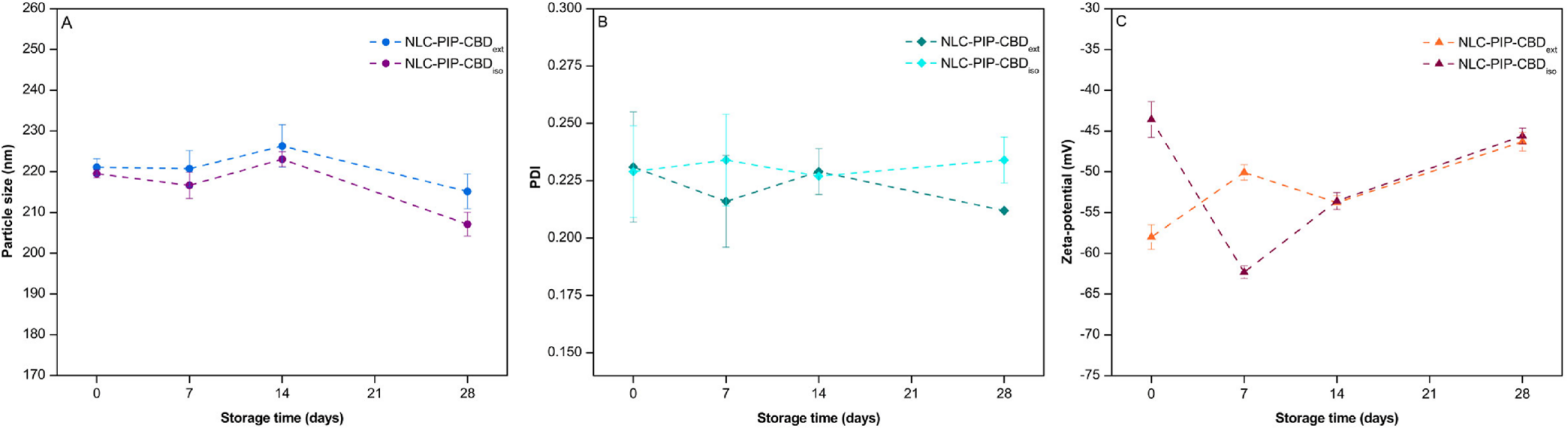
(A) Particle size expressed as z-average diameter, (B) polydispersity index, and (C) zeta potential of NLCs loaded with PIP + CBD_ext_ (NLC-PIP-CBD_ext_) and PIP + CBD_iso_ (NLC-PIP-CBD_iso_) during 28 days of storage.

Despite these variations in ZP during the storage period, the values remained above 20 mV (in modulus), indicating good physical stability. High absolute ZP values, whether positive or negative, enhance suspension stability by promoting electrostatic repulsion and reducing particle aggregation.^38^ Similar results were reported for NLCs formulated with high oleic sunflower oil (HOSO) and FHSO as lipidic constituents in different ratios, using soybean lecithin as the emulsifier.^36^

The EE of CBD and PIP in NLCs was also evaluated during the storage time to ensure their stability. The EE of CBD in both systems, NLC-PIP-CBD_ext_ and NLC-PIP-CBD_iso_, was 100% throughout the storage time, similar to that previously reported for NLCs loaded with only CBD.^17^ PIP exhibited an EE of over 99.7% for both systems during the storage period of 28 days (Table S3). The values found for PIP EE agree with those found in previous works. For example, nanoemulsions formulated with oleic acid as the lipid phase (5%) and Tween 80 (5–10%) loaded with 1 mg/mL of PIP presented an EE of 98%,^39^ while PIP-loaded NLCs prepared with pharma-grade ingredients, namely Geleol:Labrasol at a ratio of 95:5 as the lipid phase (2%) and Brij58 (3%) as a surfactant, resulted in an EE of 98.7 ± 1.4%.^40^ The disordered lipid matrix of the NLCs, along with the inclusion of liquid lipids in their composition, enhances the solubilization capacity for lipophilic compounds, such as CBD and PIP.^41^ Therefore, the results obtained in the present study indicate the high loading capacity of the developed NLCs, making them a suitable alternative for the simultaneous delivery of CBD and PIP.

### 3.2 *In Vitro* Digestion

#### 3.2.1 Droplet Size, Zeta Potential, and Morphology

The NLCs loaded with both compounds were subjected to *in vitro* digestion, and the samples obtained after each simulated phase were characterized in terms of droplet size and ZP (Figure 2). As the constituents of the NLCs are not susceptible to digestion in the mouth and no changes were observed in the microscopy images (Figure 3), the droplet size and ZP were not measured in the samples from the oral phase.

**Figure 2:**
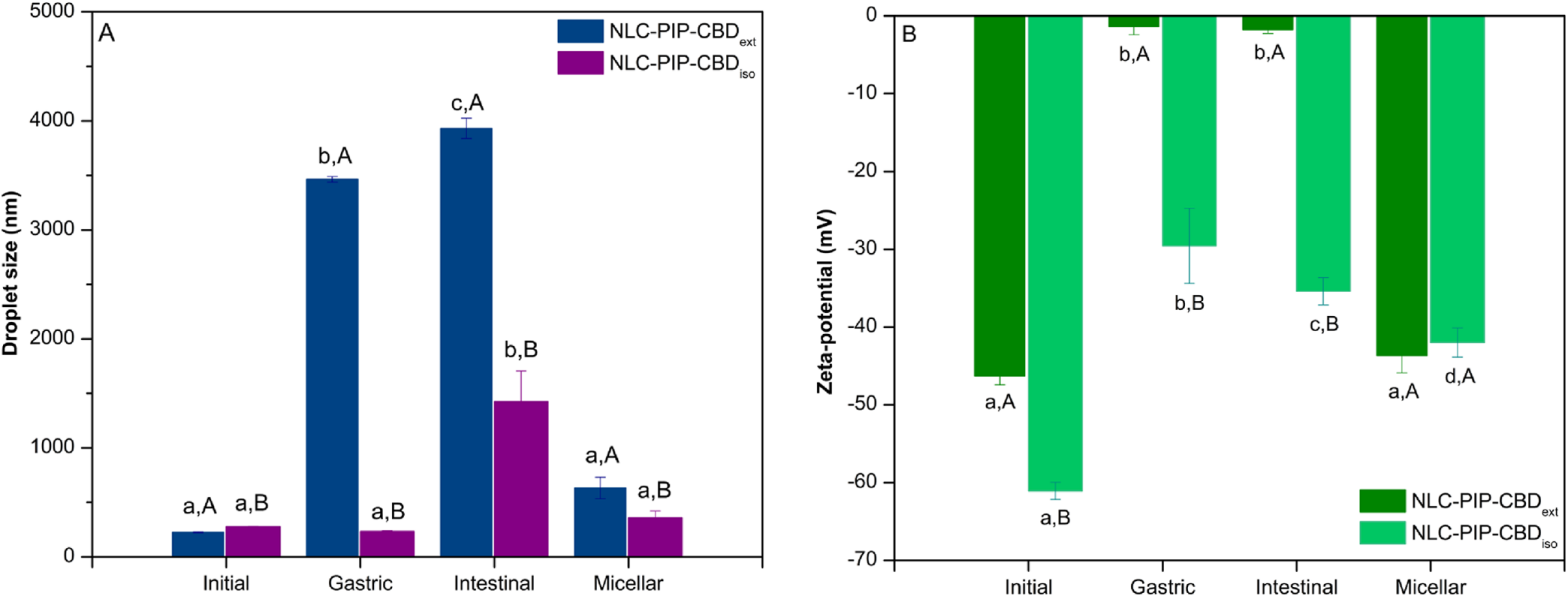
(A) Particle size expressed as z-average and (B) zeta potential of NLC-PIP-CBD_ext_ and NLC-PIP-CBD_iso_ in different stages of *in vitro* digestion. Error bars represent the standard deviation of *n* = 3 replicates. Different lower-case letters indicate significant differences between the simulated stages of *in vitro* digestion for each sample, and different upper-case letters indicate significant differences between samples at each simulated stage of *in vitro* digestion (*p* < 0.05).

**Figure 3:**
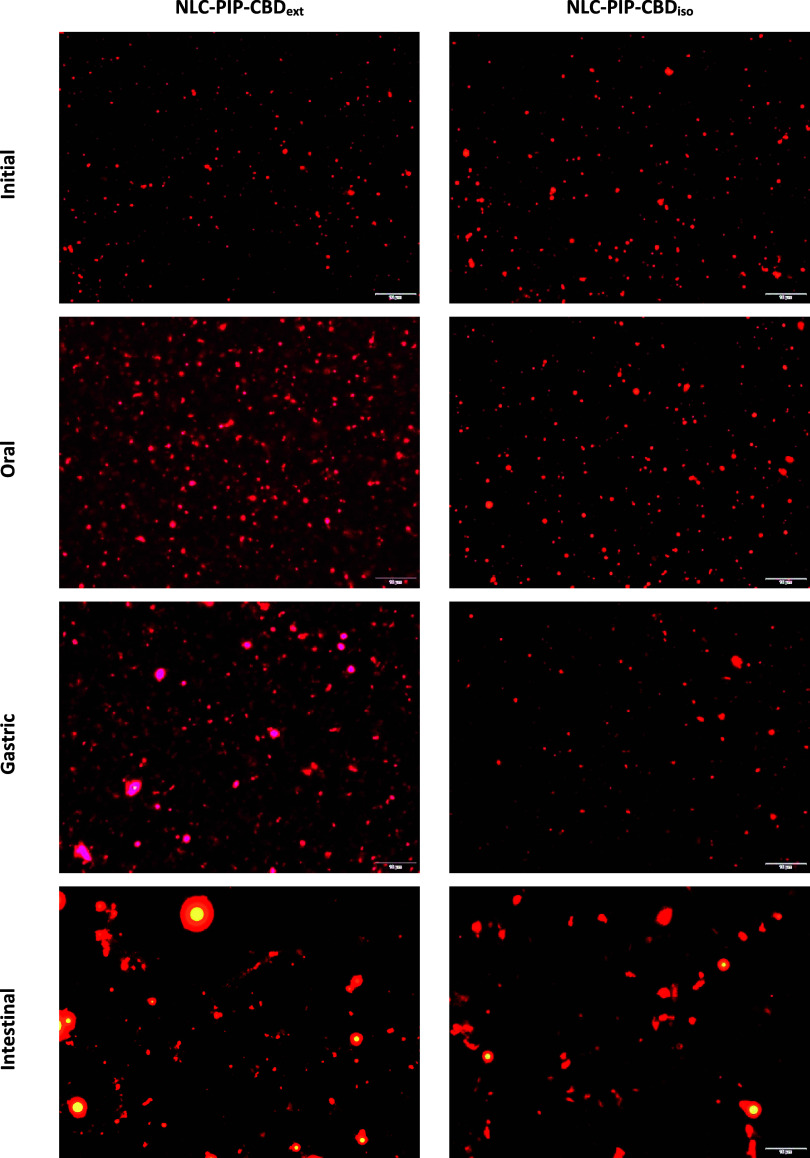
Fluorescence microscopy images of NLC-PIP-CBD_ext_ and NLC-PIP-CBD_iso_ after each stage of *in vitro* digestion. The scale bar in all images is 10 μm.

Interestingly, the CBD forms, CBD_ext_ and CBD_iso_, had a significant impact on the behavior of NLCs during *in vitro* digestion. NLC-PIP-CBD_ext_ was markedly destabilized during the simulated gastric phase, leading to a pronounced increase in the droplet size and a decrease of ZP to values close to zero, whereas NLC-PIP-CBD_iso_ showed a slight reduction in the droplet size and ZP of −29.6 ± 4.8 mV, suggesting that this formulation remained stable under gastric conditions (Figure 2). Colloidal dispersions with ZP values greater than ±20 mV are considered stable due to the electrical repulsion among particles that reduces their aggregation.^42^ A previous study evaluating the *in vitro* digestion of NLCs loaded exclusively with CBD (NLC-CBD_ext_ and NLC-CBD_iso_) also reported a minor decrease in the droplet size after the gastric phase.^32^ These findings indicate that the coincorporation of PIP with CBD_iso_ did not alter the digestion behavior of NLC-PIP-CBD_iso_, while the presence of PIP alongside CBD_ext_ significantly modified the stability of NLC-PIP-CBD_ext_ during *in vitro* digestion, suggesting that PIP interacts differently with CBD_iso_ and CBD_ext_.

Similar outcomes have been described for NLCs coloaded with PIP and other bioactive compounds, such as resveratrol (NLC-R-P) and curcumin (NLC-C-P), where distinct release profiles were observed for each system.^40^ The authors attributed these differences to several factors, including active excipient interactions, solubility of the actives in the release medium, and their spatial distribution within nanostructures. This rationale may also explain the present findings since the distinct interactions of PIP with CBD_ext_ and CBD_iso_ likely influenced their distribution within NLCs, thereby affecting their behavior during *in vitro* digestion.

The distribution of bioactive compounds in NLCs depends on their partitioning during particle formation and solubility in the lipidic matrix. In systems produced by the hot-melting technique, if the concentration of the compound in the melted lipid is well below its saturation solubility, lipid solidification results in drug distribution into the particle’s shell. Conversely, when the concentration is close to the saturation solubility, a drug-enriched core is formed.^40,43^ Accordingly, it may be hypothesized that the distinct solubility profiles of CBD_iso_ and CBD_ext_ influence their spatial distribution within the NLC matrix. Specifically, PIP-CBD_ext_ may preferentially localize at the particle surface, while PIP-CBD_iso_ is more likely to be confined to the particle core. This distribution pattern could plausibly render NLC-PIP-CBD_ext_ more susceptible to destabilization under simulated gastric conditions compared to NLC-PIP-CBD_iso_. The destabilization observed for NLC-PIP-CBD_ext_ during the gastric phase was also evident in the samples collected from both the digesta and micellar phases. After the intestinal phase, the droplet size of NLC-PIP-CBD_ext_ further increased, reaching values of approximately 4000 nm (Figure 2a), while the ZP remained close to zero (Figure 2b). In the micellar phase, which corresponds to the fraction obtained after centrifugation of the digesta sample to remove larger particles, thus mimicking the fraction that can be absorbed by intestinal epithelial cells, NLC-PIP-CBD_ext_ exhibited droplet size values of 633 ± 97 nm and a zeta potential of approximately −42 mV.

NLC-PIP-CBD_iso_ also exhibited a significant increase in the droplet size after the intestinal phase, reaching around 1500 nm, indicating destabilization at this stage, similar to the findings previously reported for NLCs loaded with only CBD.^32^ This increase in droplet size is likely associated with the formation of lipid digestion products in the small intestine, such as micelles, vesicles, and other colloidal structures.^44–47^ Importantly, the ZP of NLC-PIP-CBD_iso_ remained at −35.4 ± 1.75 mV, suggesting that the observed increase in the droplet size was not due to droplet aggregation. In the micellar phase, NLC-PIP-CBD_iso_ presented a droplet size of 359 ± 61 nm, while the ZP values were around −42 mV. The greater stability observed for NLC-PIP-CBD_iso_ during *in vitro* digestion can be associated with higher CBD bioaccessibility, as discussed in Section 3.2.3.

#### 3.2.2 FFA Release

Although NLC-PIP-CBD_ext_ and NLC-PIP-CBD_iso_ exhibited distinct behaviors during *in vitro* digestion (Figure 2), the final amounts of FFA released at the end of *in vitro* digestion were similar for both systems, with values of 22.0 ± 0.2% and 22.7 ± 0.7% for NLC-PIP-CBD_ext_ and NLC-PIP-CBD_iso_, respectively. Similar results for FFA release from both systems indicate that the interactions between the constituents of each nanostructure did not affect the triacylglycerol (TAG) hydrolysis. FFA release mainly reflects the extent of enzymatic hydrolysis and the amount of TAGs hydrolyzed, whereas particle stability during digestion is additionally influenced by the composition and reorganization of the residual lipid matrix and emulsifier coverage, where the presence of minor CBD_ext_ components may alter the postdigestion structure. In other words, two NLCs can generate comparable amounts of FFA but still differ in the structural organization of the particle,^17^ which affects its integrity, aggregation tendency, or structural collapse during and after lipolysis. For instance, Ludtke et al.^34^ observed that different amounts of FFA were released from NLCs produced with different emulsifiers (Tween 80 and soybean lecithin), which demonstrates that the NLC constituents play an important role in the extent of FFA release.

In general, a higher concentration of FFAs released during lipid digestion is associated with greater bioaccessibility of bioactive compounds, as the increased presence of lipid species in the micellar fraction enhances their micellarization.^48^ However, although the amounts of FFAs released from both NLC-PIP-CBD_ext_ and NLC-PIP-CBD_iso_ were not significantly different (*p-*value >0.05), the bioaccessibility results were strongly affected by the distinct CBD forms, CBD_ext_ and CBD_iso_. These findings suggest that the bioaccessibility of CBD and PIP loaded into the NLCs was not directly correlated with the extent of lipid digestion, as further discussed in Section 3.2.3.

The relatively low FFA release observed for NLC-PIP-CBD_ext_ and NLC-PIP-CBD_iso_ can be attributed to several structural and compositional factors, since the rate and extent of lipolysis are also influenced by formulation parameters, including lipid polymorphism, fatty acid chain length, emulsifier type, and PS.^45,49^ Particularly, the predominance of long-chain fatty acids in HSO and FHSO likely slowed down the hydrolysis kinetics, since the crystals formed by these lipids hinder lipase adsorption at the oil–water interface.^45^ Furthermore, long-chain FFAs produced during digestion have low water dispersibility and tend to accumulate at the interface, temporarily inhibiting lipase activity until they are solubilized into micelles or precipitated by calcium.^49^

#### 3.2.3 Stability, Bioaccessibility, and Estimated Bioavailability

The samples obtained from *in vitro* digestion were analyzed to assess the stability and bioaccessibility of the CBD encapsulated within the NLCs. The stability parameter refers to the proportion of each compound that remains intact and retains its active form after gastrointestinal digestion. Bioaccessibility, in turn, corresponds to the fraction of the compounds incorporated into mixed micelles, representing the portion available for absorption by intestinal epithelial cells.^31,44^

The stability of CBD did not show significant differences (*p*-value = 0.223) between NLC-PIP-CBD_ext_ and NLC-PIP-CBD_iso_ (Figure 4). The NLCs incorporated with both CBD and PIP presented increased stability for both NLC-PIP-CBD_iso_ (98 ± 4%) and NLC-PIP-CBD_ext_ (103 ± 5%) compared to the NLC-CBD_iso_ (73 ± 2%) and NLC-CBD_ext_ (83 ± 2%).^32^ In contrast, for both forms of CBD dispersed in HSO, the CBD stability was reported to be around 50%.^32^ Similarly, Cho et al.^50^ reported a retention rate of 60.4% for free PIP after the small intestine phase of *in vitro* digestion. NLCs are generally expected to enhance the stability of bioactive compounds, owing to the presence of solid lipids in their matrix, which are digested more slowly than liquid lipids.^51^ Beyond lipid composition, the stability of encapsulated compounds also depends on their affinity with the lipid species forming the NLC, the type of molecular interactions established between the compounds and particle, and the specific location of incorporation within the lipid matrix.^31^ Interestingly, although NLC-PIP-CBD_ext_ exhibited an increase in PS immediately after the gastric phase (Figure 2a), the compounds may have remained associated with the lipid components of the NLCs, thus being protected against degradation during digestion.

**Figure 4:**
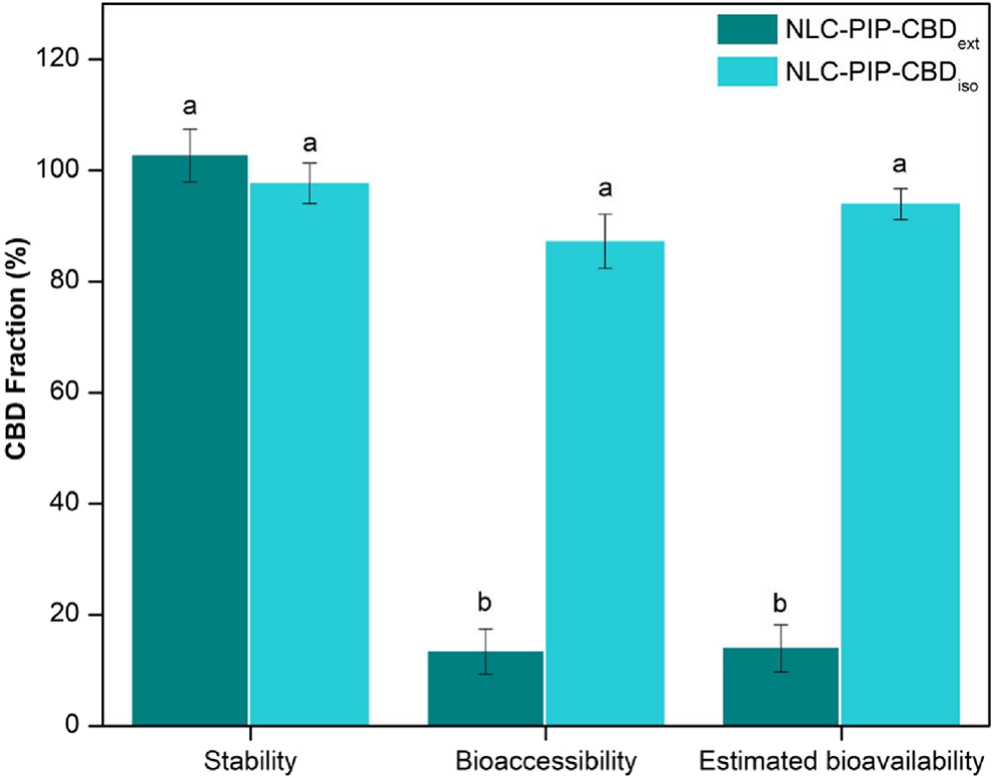
Stability, bioaccessibility, and estimated bioavailability of CBD in the nanostructures NLC-PIP-CBD_ext_ and NLC-PIP-CBD_iso_ after *in vitro* digestion. Error bars represent the standard deviation of *n* = 6 replicates. Different letters indicate significant differences between nanostructures for each parameter (*p* < 0.05).

Regarding bioaccessibility, it was observed that the NLC structures (NLC-PIP-CBD_ext_ and NLC-PIP-CBD_iso_) exhibited a marked influence (Figure 4). NLC-PIP-CBD_iso_ achieved a CBD bioaccessibility of 87 ± 5%, comparable to that observed when only CBD_iso_ was incorporated into the NLC (94 ± 5%).^32^ In contrast, NLC-PIP-CBD_ext_ significantly reduced CBD bioaccessibility, with values of only 13 ± 4%. This marked reduction may occur primarily during the transport of the compounds to the micellar fraction since the stability of the compounds was not dramatically affected by *in vitro* digestion (Figure 4). Although fluorescence microscopy images from the intestinal phase confirm the presence of mixed micelles in this system (Figure 3), these micelles may not have efficiently incorporated the bioactive compounds, which indicates that the marked reduction in bioaccessibility observed for CBD_ext_ vs CBD_iso_ may reflect a combination of formulation and digestion effects rather than a single mechanism.

In particular, CBD_ext_ contains minor cannabinoids, terpenes, and other lipophilic impurities that may alter the organization of the lipid matrix, modify the crystallinity of NLCs, and change the CBD distribution within the particles. Previous results indicated that both NLC-CBD_iso_ and NLC-CBD_ext_ presented a β-polymorphic form; however, NLC-CBD_iso_ presented more intense XRD peaks and higher melting temperatures (*T*_m_) and enthalpy changes (Δ*H*) than CBD_ext_,^17^ which may indicate distinct interaction strengths between the lipid matrix and CBD forms. During NLC formation, the nonpolar fatty acid chains align primarily through van der Waals forces, driving TAGs into ordered polymorphs.^52,53^ Because CBD_iso_ (99.2% purity) is more homogeneous than CBD_ext_, it may be integrated more readily into the lipid matrix. Generally, longer and more uniform chains facilitate stronger and more ordered crystalline packing; in contrast, shorter, heterogeneous mixtures promote imperfect crystallization.^54^ These variations alter the viscosity of the molten lipid phase and crystallization rate, which are crucial determinants of particle nucleation and growth.

At the same time, during gastrointestinal digestion, the additional components present in CBD_ext_ can also influence the assembly and solubilization capacity of the mixed micelles, which have a limited hydrophobic core volume for bioactive integration. Thus, the presence of additional lipophilic species can lead to competitive micellar solubilization, where components with higher lipophilicity or distinct molecular geometries compete with CBD for integration into these limited hydrophobic domains. This competition can lead to the saturation of the micellar phase, effectively reducing the micellarization efficiency of CBD by limiting its transfer from the oil droplets to the aqueous intestinal environment.^55^

The stability and bioaccessibility data can be used to provide an estimate of the bioavailability of the compounds, defined as the portion that can potentially be absorbed by the intestinal epithelial cells.^56^ As expected, the estimated bioavailability followed the same behavior observed for bioaccessibility: NLC-PIP-CBD_ext_ exhibited significantly lower values for CBD (*p*-value = 0.002) compared with NLC-PIP-CBD_iso_ (Figure 4). It should be emphasized, however, that this *in vitro* estimation has inherent limitations, as it does not account for the complex metabolic reactions occurring *in vivo* that strongly affect the intestinal absorption of the compounds.^29,31^ A more accurate determination of bioavailability requires *in vivo* studies, in which plasma concentrations are measured in animals or humans after administration of a known dose of a specific compound. This approach enables the evaluation of the fraction of the compound that undergoes digestion, absorption, metabolism, and ultimately reaches the systemic circulation.^57^ Nonetheless, *in vitro* estimations remain a valuable tool in food and nutraceutical research, as they provide early insights into formulation performance and guide the design of delivery systems with improved potential for enhancing bioavailability *in vivo*.

### 3.3 *In Vivo* Pharmacokinetics

The absolute oral bioavailability of free CBD is approximately 6–13%,^22^ mainly ascribed to poor water solubility and extended first-pass metabolism, where CBD undergoes monohydroxylation at C-7, forming the 7-OH metabolite, mediated by CYP450 enzymes, specifically CYP3A4 and CYP2C19.^11,13,58^ However, it has been reported that the coingestion of CBD with lipid species can significantly enhance its bioavailability, as compared with the fasted state.^59^ This is attributed to lipid digestion and the formation of mixed micelles, which can enhance the dissolution rate and solubility of lipophilic compounds, as well as lymphatic absorption when long-chain fatty acids are involved.^11^ However, the inconsistency in meal times, food composition, lipid type, and proportion can result in variable bioavailability, representing a limitation of the administration of CBD solely with foods.^13^ To overcome these issues, the incorporation of CBD into lipid formulations consisting of long-chain fatty acids has demonstrated promising results in the mitigation of the effects of food on the absorption of CBD.^22,29^ Additionally, Cherniakov et al.^22,60^ demonstrated that an SNEDDS formulation containing CBD and the bioenhancer PIP was able to enhance CBD bioavailability, probably through the inhibition of Phase I and Phase II metabolism.

Thus, we conducted an *in vivo* pharmacokinetic study to evaluate the effect of simultaneous delivery of CBD and PIP coencapsulated within an NLC on CBD bioavailability. Based on the *in vitro* results, only CBD_iso_ was selected for this study. Figure 5 presents the plasma concentration of CBD_iso_ vs. time profiles of HSO-CBD_iso_, NLC-CBD_iso_, and NLC-PIP-CBD_iso_ following their oral administration to male and female mice. Table 1 summarizes the corresponding pharmacokinetic parameters. As previously mentioned, the gender effect on CBD pharmacokinetics was evaluated because it was hypothesized that the increase in CBD bioavailability through its incorporation into advanced delivery systems could reduce the gender-based pharmacokinetic variability.^16^

**Figure 5:**
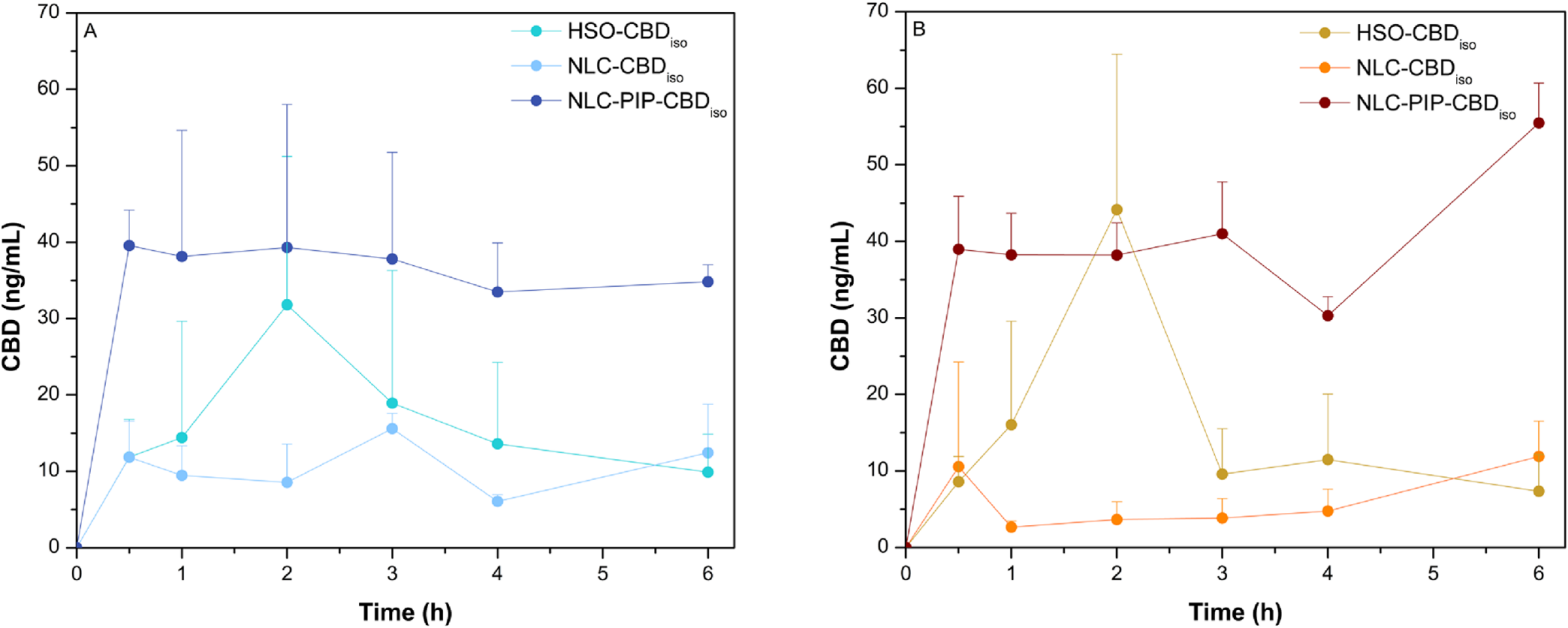
Plasma concentration–time profiles of CBD (mean ± SD) in (A) male and (B) female mice following a single oral administration of CBDiso, NLC-CBDiso, and NLC-PIP-CBDiso formulations, each corresponding to a 1 mg CBD dose (*n* = 5 per group).

**Table 1:** Pharmacokinetic Parameters Obtained Following Single-Dose Oral Administration of CBD_iso_, NLC-CBD_iso_, and NLC-PIP-CBD_iso_ to Male and Female Mice, Corresponding to 1 mg CBD^a^

	CBD_iso_		NLC-CBD_iso_		NLC-PIP-CBD_iso_	
	male	female	male	female	male	female
AUC_0–6h_ (h ng/mL)	94.7	92.5	55.7	31.2	200.6	218.5
*t*_max_ (h)	2.0	2.0	3.0	6.0	0.5	6
*C*_max_ (ng/mL)	31.8 ± 19.4^a,AB^	44.2 ± 20.3^a,A^	15.6 ± 2.0^a,B^	11.9 ± 4.6^a,B^	39.6 ± 4.6^b,A^	55.5 ± 5.2^a,A^

a Different lower-case letters indicate significant differences between male and female mice (*n* = 5) for the same sample; different upper-case letters indicate significant differences between the samples for the same gender (*p* < 0.05).

The CBD plasma concentration curves of HSO-CBD_iso_ presented a similar profile for both male and female mice, with *t*_max_ at 2 h (Figure 5). However, although not statistically significantly different (*p*-value = 0.354), the *C*_max_ of HSO-CBD_iso_ was higher in female than in male mice (Table 1), corroborating the previous findings reported in studies that evaluated the gender effect in human volunteers.^15,16^ The higher systemic exposure observed in females is often attributed to sexually dimorphic expression of CYP450, particularly the CYP3A family and CYP2C19, which are the primary drivers of CBD metabolism. Furthermore, the higher proportion of adipose tissue in females may serve as a deeper distribution reservoir for lipophilic CBD, while differences in gastric emptying rates and lower glomerular filtration could further prolong system retention.^61^ Interestingly, the sex-dependent differences observed for the *C*_max_ of HSO-CBD_iso_ were mitigated in the NLC-CBD_iso_ group (*p*-value = 0.133). This aligns with the “normalization” effect reported by Knaub et al.^16^ for an SEDDS-CBD formulation, suggesting that high-efficiency lipid-based delivery systems (like NLCs or SEDDS) can bypass traditional rate-limiting physiological barriers, such as the gastric transit time and first-pass metabolism, thereby reducing intersex variability.

The administration of NLC-PIP-CBD_iso_ resulted in a significant *C*_max_ increase compared to NLC-CBD_iso_ in both male and female mice (*p*-value <0.001). This effect may be partially associated with the reported bioenhancing properties of PIP, including the modulation of P-gp transport activity and CYP450-mediated metabolism.^24,62^ In addition to possible metabolic- and transporter-related effects, the increased systemic exposure may also be influenced by formulation-dependent factors, including improved CBD solubilization, enhanced intestinal absorption, and lipid-mediated transport mechanisms. PIP has also been reported to enhance drug absorption by modifying the dynamics and permeability of the biological membranes.^63^ Due to its apolar nature, PIP can partition into the lipid core and facilitate the permeation of associated compounds.^63^ Furthermore, PIP has been associated with increased membrane fluidity, elongation of microvilli, and an increase of the absorptive surface of the intestine.^63,64^ Supporting this hypothesis, Raghunath et al.^62^ investigated the influence of PIP on the intestinal permeation, pharmacodynamics, and pharmacokinetics of insulin-loaded chitosan-coated solid lipid nanoparticles (Ch-SLNs) in rats and observed enhanced permeation across different intestinal segments, along with sustained pharmacological effects compared with subcutaneous insulin administration. Collectively, these findings suggest that the increased CBD exposure observed for NLC-PIP-CBD_iso_ may result from a combination of formulation-related and bioenhancing effects of PIP, although the precise mechanisms remain to be elucidated.

The *C*_max_ value of NLC-CBD_iso_ was lower than that of HSO-CBD_iso_ in both male and female mice (Table 1). The lipid digestion rate is largely determined by the surface area available for enzyme interaction, as well as the ability of digestive enzymes to adsorb onto the oil–water interface and then access TAG molecules to initiate lipolysis.^65^ The FFA released during lipolysis, along with bile salts and phospholipids, contributes to the formation of mixed micelles. Bioactive compounds, such as CBD, are initially solubilized in the lipid phase and then incorporated into these mixed micelles, which transport them to the epithelial cells of the small intestine.^66,67^ As the formation of mixed micelles occurs at the same rate as lipid digestion, differences in lipolysis kinetics between formulations may directly influence the systemic exposure of bioactive compounds.^48^

In this context, the presence of the solid lipid (FHSO) and the emulsifier in the NLC-CBD_iso_ structure may reduce the rate of lipolysis compared to HSO-CBD_iso_, where the lipid phase remains more accessible to enzymatic action.^32^ Another possible explanation for the different performances between NLC-CBD_iso_ and HSO-CBD_iso_ may involve a mismatch between the CBD release from the NLC matrix and the rate of lipid digestion. If CBD remains partially entrapped within the solid lipid core during digestion, then its transfer into mixed micelles could be limited, even in the presence of ongoing lipolysis. Such a release–digestion mismatch has been reported to reduce the bioaccessibility of lipophilic compounds in structured lipid systems.^45^ Therefore, the lower *C*_max_ observed for NLC-CBD_iso_ likely reflects a combination of factors, including altered lipolysis kinetics and potential limitations in drug release, which warrants further investigation through dedicated *in vitro* digestion and release studies.

The AUC_0–6h_ presented the same trend observed for *C*_max_, with the lowest values obtained for NLC-CBD_iso_ in both male and female mice. However, for this parameter, NLC-PIP-CBD_iso_ exhibited values 2.1- and 2.4-fold higher than those of HSO-CBD_iso_ in male and female mice, respectively, corroborating the hypothesis that the incorporation of an absorption enhancer into the NLC can result in an advanced simultaneous delivery system capable of increasing the bioavailability of lipophilic compounds, such as CBD. For an SNEEDS formulation containing CBD and PIP, Cherniakov et al.^22^ observed a 2-fold increase in the AUC compared to SNEDDS containing only CBD, and a 6.3-fold increase compared to CBD vehiculated in a mixture composed of propylene glycol/ethanol/water (4.5:4.5:1, v/v), both administered to male Wistar rats. Interestingly, these authors observed that intestinal, rather than hepatic, Phase I and Phase II metabolism processes have a prominent contribution to the first-pass effect of poorly water-soluble and highly metabolized compounds. Their findings indicate that the effective inhibition of intestinal metabolism by the utilization of bioenhancers such as PIP is achieved only if it reaches the enterocyte surface in its soluble state. This means that the incorporation of CBD and PIP into lipid-based delivery systems exerts two functions simultaneously: inhibiting the first-pass metabolism and delivery of these poorly water-soluble molecules to the enterocyte surface in their solubilized state.^22^ These findings agree with our *in vitro* results, where NLC promoted an enhanced CBD stability during the *in vitro* digestion in comparison with HSO-CBD_iso_, thereby resulting in a higher bioaccessibility.^32^

However, in the present study, no correlation was observed between the bioavailability estimated from *in vitro* digestion and *in vivo* bioavailability (*r* ^2^ = 0.22). While NLC-PIP-CBD_iso_ showed high values in both models, NLC-CBD_iso_ showed high *in vitro* bioavailability but failed to improve *in vivo* absorption compared with oil (Figure 6). This discrepancy suggests that *in vitro* data alone may not adequately predict the *in vivo* performance of structured lipid systems, particularly when additional physiological absorption mechanisms are involved. Establishing a reliable *in vitro*–*in vivo* correlation (IVIVC) for lipid-based formulations remains challenging because the oral absorption of highly lipophilic compounds depends not only on micellarization during digestion but also on intestinal permeability, enterocyte uptake, chylomicron formation, lymphatic transport, and first-pass metabolism.^29,68^ In particular, CBD is a highly lipophilic compound that may undergo intestinal lymphatic transport following incorporation into chylomicrons, which are lipoproteins assembled in enterocytes in the presence of long-chain fatty TAGs.^69^ This pathway is strongly influenced by the lipid composition and digestion behavior and is not reproduced by static *in vitro* digestion models.^57^ Therefore, formulations exhibiting similar *in vitro* bioaccessibility may still differ substantially in systemic exposure *in vivo* due to differences in postabsorptive processing and lymphatic uptake.

**Figure 6:**
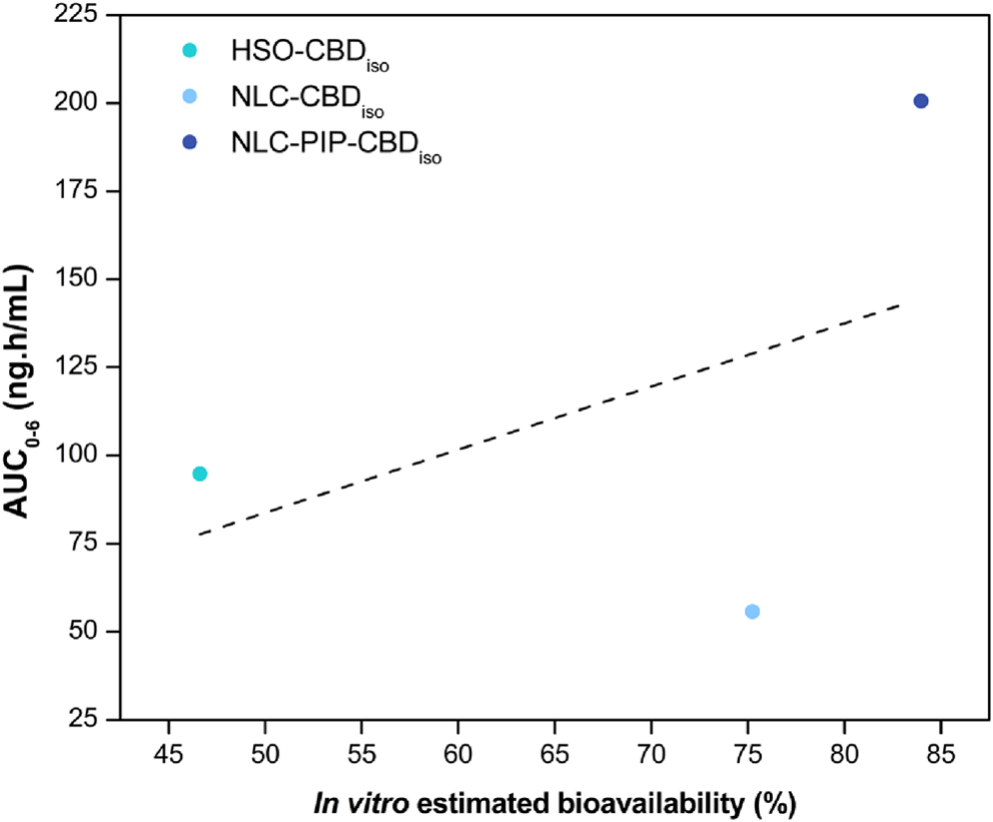
Correlation between the *in vitro* estimated bioavailability of CBD and AUC_0–6h_ of CBD plasma concentration–time profiles following oral administration of HSO-CBD_iso_, NLC-CBD_iso_, and NLC-PIP-CBD_iso_.

Additionally, although the presence of long-chain TAGs in the lipid matrix of NLCs contributes to the incorporation of CBD into chylomicrons, favoring lymphatic uptake,^29,70^ solid FHSO may have contributed to the poor IVIVC observed. Highly saturated solid lipids, such as FHSO, can alter lipid digestion kinetics, drug release behavior, and structural reorganization of lipid particles during digestion.^71^ Although NLC-CBD_iso_ exhibited high *in vitro* micellarization, the presence of the solid lipid may have limited CBD release, delayed transfer into mixed micelles, or impaired subsequent uptake and chylomicron incorporation *in vivo*.^45,71^ These effects are not fully captured by static *in vitro* digestion protocols, which primarily evaluate lipolysis and micellar solubilization but do not simulate intestinal transport or metabolic processes.^57^

Similar limitations of IVIVC for lipid-based formulations have been previously reported. For instance, a study evaluating eight lipophilic compounds formulated in lipid-based delivery systems established a linear IVIVC for only two compounds (danazol and griseofulvin).^72^ De Prá et al.^29^ observed no clear IVIVC between in vitro digestion and the in vivo bioavailability of CBD lipid formulations. Collectively, these findings reinforce the importance of complementary *in vivo* studies for evaluating lipid-based nanocarriers intended for the oral delivery of highly lipophilic compounds.

In conclusion, this work demonstrated the potential of food-grade NLCs to codeliver two lipophilic compounds, CBD and PIP, with high EE and excellent stability over 28 days of storage. Additionally, the results indicate that the forms of CBD, CBD_iso_ or CBD_ext_, did not affect the physicochemical characteristics of NLCs in the presence of PIP. However, the CBD form had a marked effect on the *in vitro* digestion behavior of the nanostructures, where NLC-PIP-CBD_ext_ presented significantly lower CBD bioaccessibility in comparison with NLC-PIP-CBD_iso_. These findings indicate that the form in which CBD is vehiculated can strongly affect the way it interacts with the lipid matrix as well as with the bioenhancer PIP, ultimately affecting its absorption after oral ingestion. Interestingly, the *in vivo* pharmacokinetic study demonstrated that NLC–CBD_iso_ did not enhance the CBD_iso_ absorption as compared to HSO–CBD_iso_, contradicting the results of the *in vitro* digestion evaluation, which reinforces the necessity of conducting *in vivo* validation studies to accurately state the role of novel nanostructures on the bioavailability of bioactive compounds. This particular behavior may be associated with the combination of two factors: (i) the HSO used as the carrier oil in the control sample (HSO-CBD_iso_) is rich in long-chain fatty acids, which may favor the lymphatic absorption of CBD_iso_; and (ii) the presence of solid lipid FHSO in the NLC lipid matrix may have slowed down its lipolysis rate during digestion, thereby decreasing the systemic exposure of CBD_iso_ delivered through this nanostructure. In contrast, NLC-PIP-CBD_iso_ showed a 2-fold higher AUC compared to HSO–CBD_iso_, which confirmed the hypothesis that coadministration of CBD with a bioenhancer can increase its absorption after oral ingestion. Additionally, the results of this study demonstrated that gender had no significant effect on the AUC, which suggests that NLC-PIP-CBD_iso_ can mitigate interindividual variations in CBD absorption. Overall, this study demonstrates the potential of coadministration of CBD with bioenhancers vehiculated in NLCs to improve CBD absorption, emphasizing the importance of appropriate dose standardization to ensure consistent and effective delivery.

### 3.4 Study Limitations

A limitation of the present study is that the pharmacokinetic study was restricted to a 6 h sampling period and a relatively sparse sampling design. This experimental design was selected based on a previous report describing the relatively rapid oral absorption of CBD in mice;^29^ however, it may not have been sufficient to fully characterize the terminal elimination phase or the delayed absorption processes associated with the formulations investigated. Consequently, the pharmacokinetic analysis was limited to AUC_0–6h_ rather than AUC_0‑∞_, which may underestimate the total systemic exposure and potentially overlook formulation-dependent differences at later time points. In particular, because PIP has been reported to modulate CBD metabolism through the inhibition of metabolic enzymes, the current sampling window may have underestimated the potential differences in CBD half-time and systemic exposure among the treatment groups. Furthermore, the hypothesized contribution of intestinal lymphatic transport following the administration of the formulations was not directly assessed. Since lymphatic absorption is often associated with a delayed *T*_max_ and prolonged absorption phases, additional late sampling time points would be necessary to better characterize the contribution of this pathway to CBD bioavailability.

Although the sampling schedule was sufficient to characterize the initial absorption phase and compare early exposure among formulations, a denser sampling strategy would improve the robustness of pharmacokinetic parameter estimation, particularly for parameters associated with the terminal phase. Therefore, the pharmacokinetic differences observed in the present study should be interpreted within the constraints of the experimental design.

An additional limitation of this study is the absence of a free CBD + free PIP control group, which does not allow complete discrimination between the pharmacokinetic effects arising from PIP itself and those associated with the codelivery of CBD and PIP within the NLC system. Therefore, although the results demonstrate that NLC-PIP-CBD_iso_ altered CBD pharmacokinetics, the relative contributions of PIP-mediated bioenhancement and formulation-dependent effects cannot be fully resolved. Future studies incorporating free compound combinations, along with mechanistic absorption and metabolism experiments, would help clarify the individual and synergistic contributions of PIP and the NLC system.

## Supplementary Material



## Data Availability

The data is available in the DataRepositoriUM: 10.34622/datarepositorium/KV3JCU.

## Notes

Additional experimental details, including the composition of simulated digestive fluids, protocol for blood collection from mice, and additional results of entrapment efficiency of piperine into the NLCs (PDF)
