# Supplementary material for: Effect of Piperine Codelivery on the Oral Bioavailability of Cannabidiol: Insights from In Vitro Digestion and In Vivo Pharmacokinetics
Source: J Agric Food Chem. 2026 Jun 18;74(25):19807–19. doi: 10.1021/acs.jafc.6c02678 (PMC13329993; doi:10.1021/acs.jafc.6c02678)
Supplement: Supplementary file 1 [file jf6c02678_si_001.pdf]

Supporting information

Effect of piperine co-delivery on the oral bioavailability of cannabidiol: Insights from *in vitro* digestion and *in vivo* pharmacokinetics

Renata Vardanega[1,2]*, Fernanda L. Lüdtke[1,2], Luis Loureiro[1,2], Andrea Fernández-Carrera[3], Joana Santos[1], Armando Venâncio[1,2], Ana C. Pinheiro[1,2], África González-Fernández[4,5], António A. Vicente[1,2]

[1]Centre of Biological Engineering, University of Minho, Braga, 4710-057, Portugal.

[2]LABBELS - Associate Laboratory, Guimarães, 4800-058, Portugal.

[3]CINBIO, University of Vigo, Vigo, 36310, Spain.

[4]CINBIO, Immunology group, University of Vigo, Vigo, 36310, Spain.

[5]Immunology, Instituto de Investigación Sanitaria Galicia Sur (IIS-GS), SERGAS-UVIGO, Vigo, 36312, Spain.

*Corresponding author: renata.vardanega@ceb.uminho.pt + 351 253 601 962

Table S1: Composition (mmol/L) of simulated digestive fluids according to recommended by Brodkorb et al. [*Nature Protocols*, 14, (2019) p. 991–1014].

**Salt**	**Simulated salivary fluid (SSF)**	**Simulated gastric fluid (SGF)**	**Simulated intestinal fluid (SIF)**
KCl	15.1	6.9	6.8
KH_2PO_4	3.7	0.9	0.8
$NaHCO_3$	13.6	25.0	85.0
NaCl	-	47.2	38.4
$MgCl_2(H_2O)_6$	0.15	0.12	0.33
$(NH_4)_2CO_3$	0.06	0.5	-
$CaCl_2(H_2O)_2$	1.5	0.15	0.6

Table S2: Protocol for blood collection via mandibular vein.

	**Mice code**									
**Collection time (h)**	**1**	**2**	**3**	**4**	**5**	**6**	**7**	**8**	**9**	**10**
0										
0.5										
1										
2										
3										
4 (cardiac punch)										
6 (cardiac punch)										

Table S3: Entrapment efficiency of PIP into the NLCs during 28 days of storage at 4 °C.

Sample	Day 0	14 days	28 days
NLC-PIP-CBD_{ext}	99.7 ± 0.3	99.80 ± 0.03	99.80 ± 0.01
NLC-PIP-CBD_{iso}	99.7 ± 0.2	99.82 ± 0.02	99.80 ± 0.01
